# Coexistence of Cornual Pregnancy and Placenta Increta: A Report of a Rare Case

**DOI:** 10.7759/cureus.94201

**Published:** 2025-10-09

**Authors:** Mariana Quilhó Pereira, Inês Tlemçani, Joana Almeida-Tavares, Alexandre Valentim-Lourenço, Luísa Pinto

**Affiliations:** 1 Department of Obstetrics and Gynecology, Hospital de Santa Maria, Unidade Local de Saúde de Santa Maria, Lisbon, PRT; 2 Department of Obstetrics, Gynecology and Reproductive Medicine, Hospital de Santa Maria, Unidade Local de Saúde de Santa Maria, Lisbon, PRT; 3 Department of Pathology, Hospital de Santa Maria, Unidade Local de Saúde de Santa Maria, Lisbon, PRT

**Keywords:** cornual pregnancy, hysterectomy, placenta accreta spectrum, placenta increta, ultrasonography

## Abstract

Placenta accreta spectrum (PAS) comprises abnormal placental adherence to the uterine wall, typically associated with prior cesarean or other uterine surgery. Cornual pregnancies are a rare form of ectopic gestation, associated with a high risk of uterine rupture.

We describe the case of a 37-year-old woman with no prior uterine surgeries, who was diagnosed with a cornual pregnancy associated with placenta increta. Ultrasound findings included fetal malformations and signs of placental invasion. After fetal demise and termination, surgical exploration revealed a uterine rupture at the right cornua. The histopathological examination confirmed a cornual pregnancy with placenta increta. This case underscores the importance of maintaining a high index of suspicion for PAS in atypical clinical contexts, particularly when coexisting with cornual pregnancy, due to the elevated risk of uterine rupture.

## Introduction

Placenta accreta spectrum (PAS) disorders are characterized by abnormal placental invasion into the myometrium due to defective decidualization [1,2]. The incidence of PAS has increased in parallel with rising cesarean delivery rates, but non-cesarean risk factors such as uterine curettage, in vitro fertilization (IVF), advanced maternal age, and multiparity are also implicated [3,4].

Cornual pregnancies are a rare and potentially life-threatening form of ectopic pregnancy, accounting for 2%-4% of all ectopic gestations [5]. Implantation occurs within the uterine horn, which is surrounded by a thin myometrial layer, predisposing to rupture and massive hemorrhage [6]. The simultaneous occurrence of PAS and cornual pregnancy is exceptionally rare and, to the best of our knowledge, has only been described once in the literature [7]. We report a case of this coexistence, successfully managed through surgical intervention.

## Case presentation

**Figure 1:**
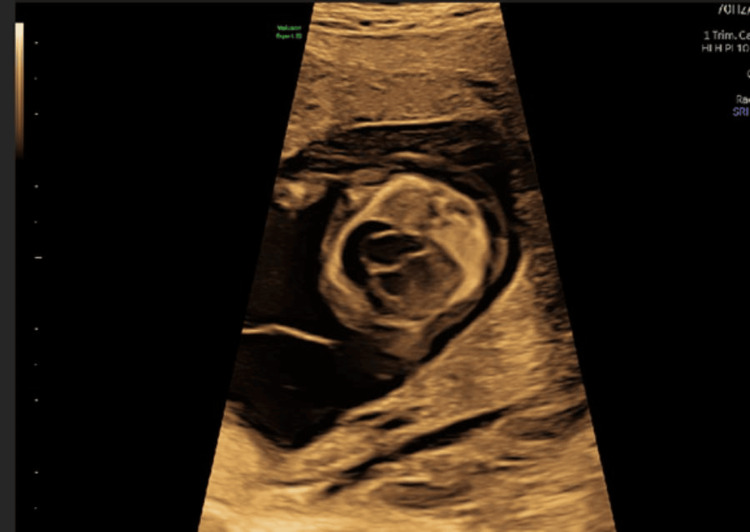
First-trimester ultrasound demonstrating cardiomegaly and cardiac malformation that is difficult to characterize, and pericardial effusion.

A 37-year-old woman, gravida 5 para 3, with a history of three prior vaginal deliveries and one molar pregnancy requiring uterine curettage, was referred to our fetal medicine unit for further assessment following abnormal first-trimester ultrasound findings. At 13 weeks and 4 days’ gestation, transabdominal ultrasound revealed a hydropic fetus with generalized edema, pericardial effusion, enlarged jugular lymphatic sacs, and a suspected cardiac anomaly-possibly Ebstein anomaly, as shown in Figure 1.

**Figure 2:**
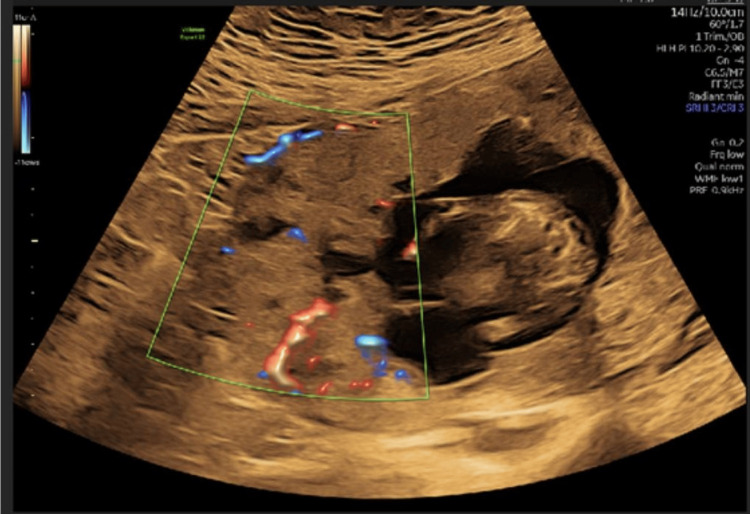
First-trimester ultrasound demonstrates the lack of interface between the placenta and the myometrium, and a serosa composed of abundant placental lakes and increased vascularity.

The placenta appeared fundal and markedly thickened. There was no identifiable interface between the placenta and the myometrium, with numerous placental lacunae and increased vascularity extending toward the uterine serosa, as demonstrated in Figure 2. Additional findings included two amniotic bands and a single umbilical artery.

**Figure 3:**
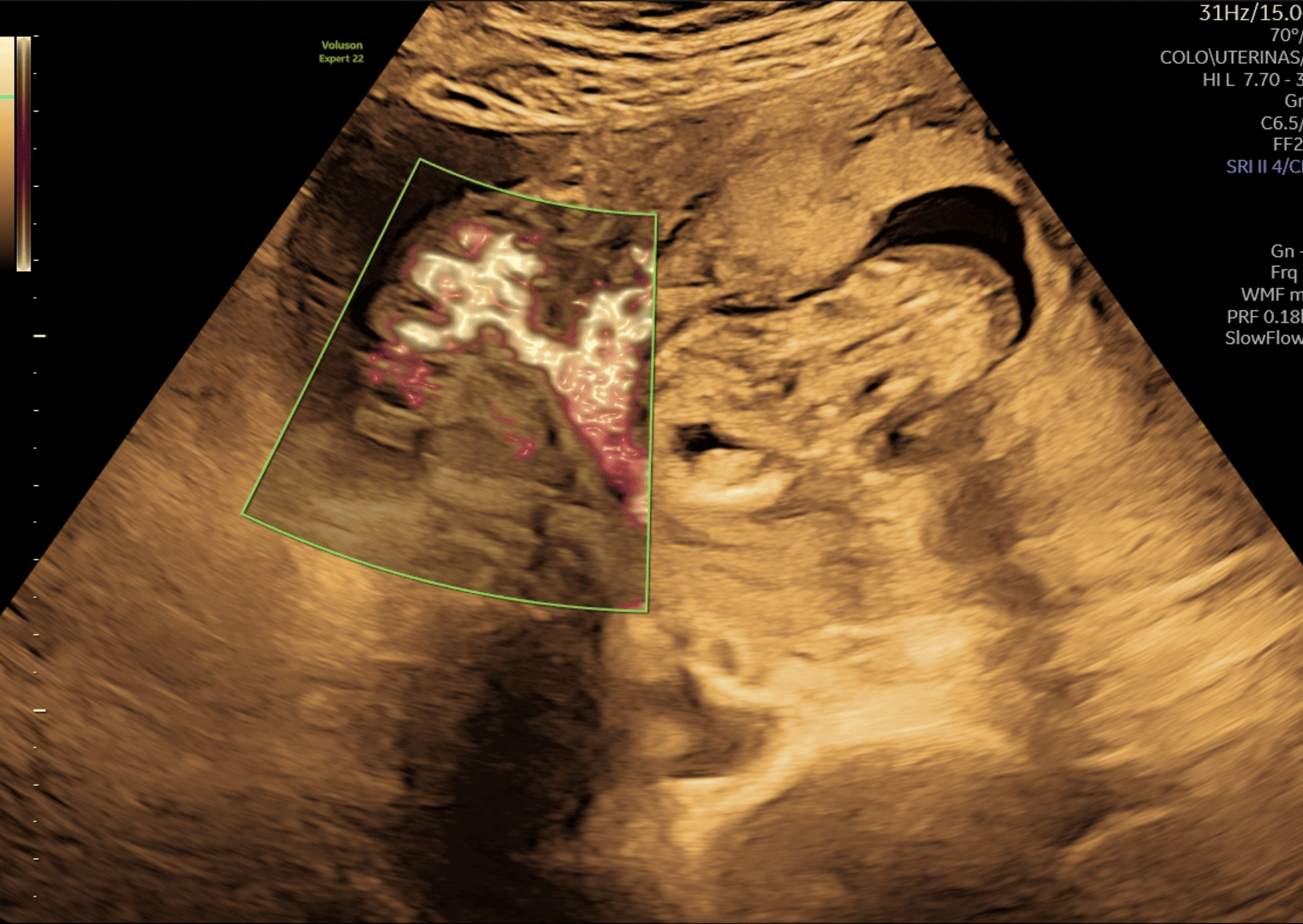
Free pelvic fluid suggestive of hemoperitoneum and increased vascularity at the placental insertion site extending beyond the uterine serosa.

Following counseling on the poor fetal prognosis, the patient elected for pregnancy termination. Amniocentesis and feticide were performed during the same procedure. Quantitative fluorescent polymerase chain reaction (QF-PCR) and comparative genomic hybridization (CGH) array returned normal results. A follow-up ultrasound revealed free pelvic fluid suggestive of hemoperitoneum and increased vascularity at the placental insertion site extending beyond the uterine serosa (Figure 3).

Despite these findings, the patient remained hemodynamically stable and asymptomatic, and surgical intervention was scheduled for three days later due to the availability of an experienced medical team. Informed medical consent for hysterectomy was given to the patient and signed before surgery.

Intraoperatively, multiple blood clots were observed in the abdominal cavity, and a uterine rupture was identified at the right cornua, with partial extrusion of the fetus through the defect. The fallopian tubes, ovaries, omentum, and peritoneum were macroscopically normal. An intrafascial hysterectomy was performed. Estimated blood losses were 400 mL, with no intraoperative complications.

**Figure 4:**
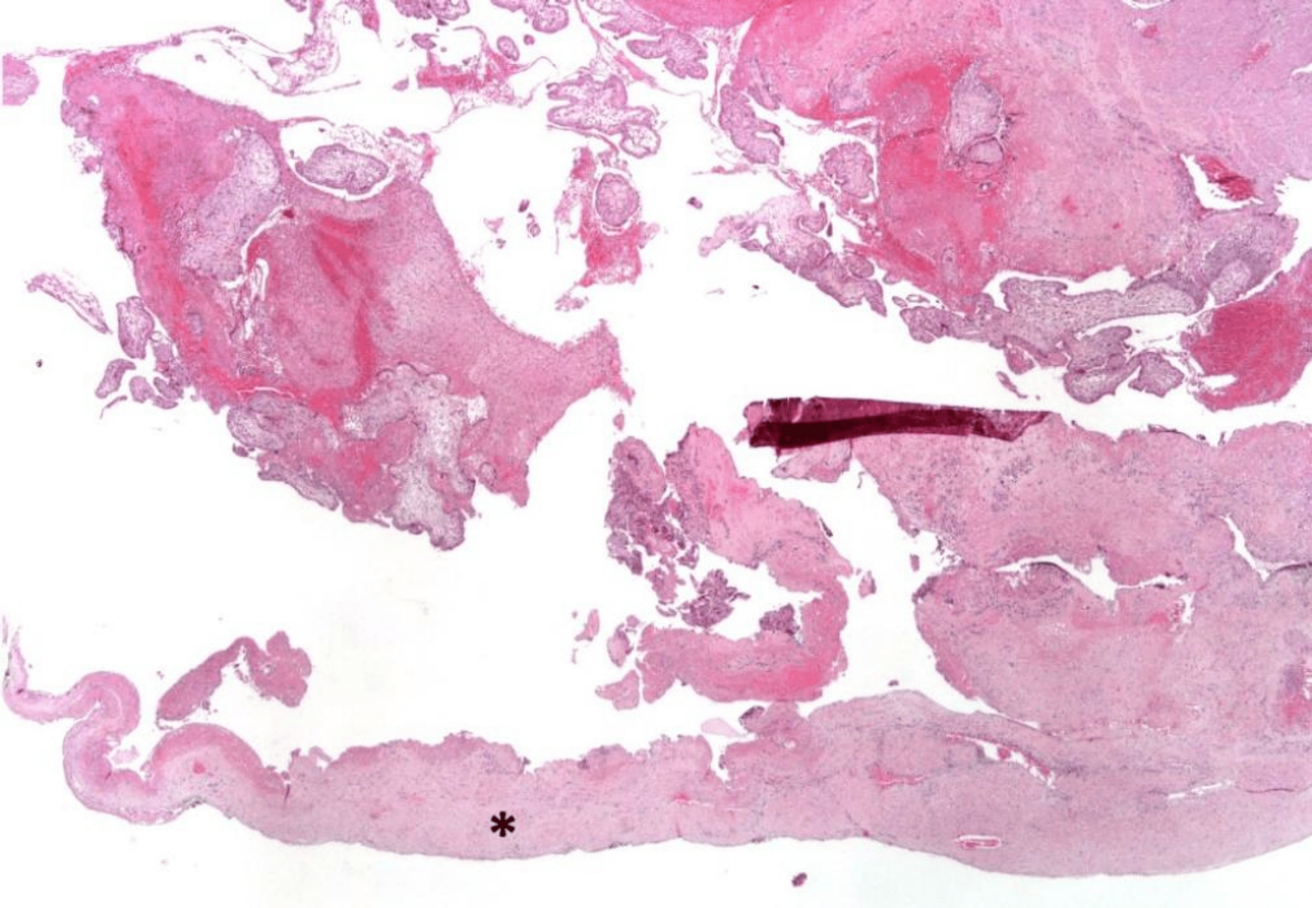
Low magnification of the uterine wall rupture area: notice the marked decrease in thickness of myometrium (*), hematoxylin and eosin (H&E) stain 20x amplification.

The histopathological examination of the fetus revealed features consistent with 13 to 14 weeks’ gestation, including facial and limb dysmorphisms. The internal organ development was appropriate for gestational age, and the gonads were consistent with a male sex fetus. Examination of the uterus revealed an adherent placental tissue to the right cornua, with extensive necrosis and a significant thinning of the myometrial wall (Figure 4).

**Figure 5:**
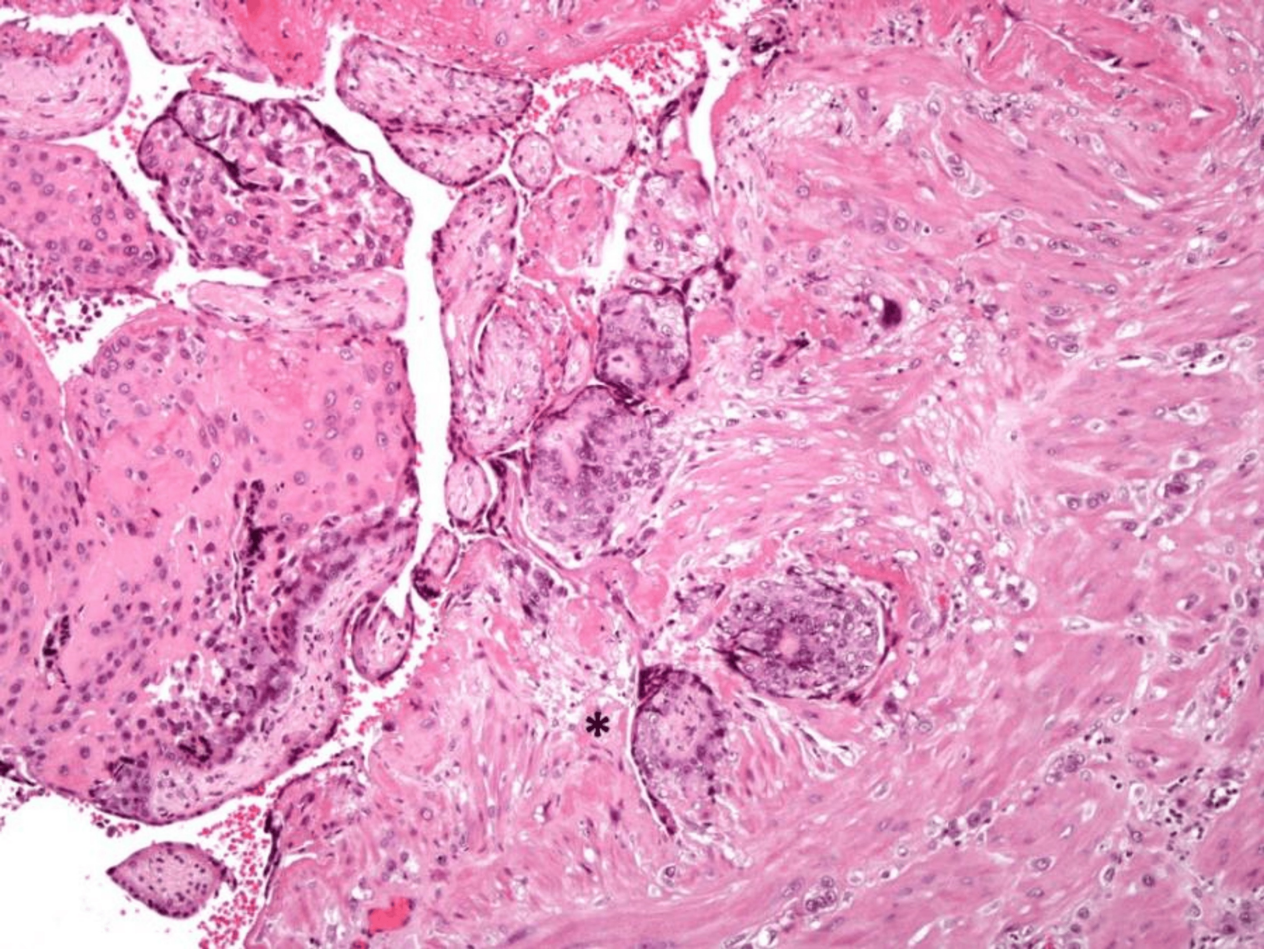
Higher magnification of the placental abnormal interface with the uterine wall–there is villous tissue in direct contact with smooth muscle fibers of the myometrium, without interposition of decidua and fibrin (*), hematoxylin and eosin (H&E) stain 100x amplification.

Chorionic villi were infiltrating the myometrium, and the uteroplacental interface appeared to be irregular (Figure 5). A focal discontinuity in the uterine wall was also present. The endometrium, cervix, and adnexa appeared normal.

These findings confirmed the diagnosis of placenta increta involving a cornual ectopic pregnancy.

## Discussion

PAS is most commonly associated with prior uterine surgery, particularly cesarean section. However, non-surgical risk factors including uterine curettage, multiparity, and maternal age over 35 have also been reported [2-4]. In this case, despite the absence of a cesarean delivery, the patient had multiple risk factors for abnormal placentation.

Cornual pregnancies, even though they are rare, are associated with a delayed diagnosis and a high risk of uterine rupture due to the distensibility of the interstitial myometrium [5-8]. The coexistence of PAS within a cornual pregnancy creates a high-risk scenario for massive hemorrhage and uterine rupture, often necessitating emergent hysterectomy [9].

Ultrasound remains the primary diagnostic method for both PAS and cornual pregnancies. PAS is typically characterized by loss of a clear zone, presence of placental lacunae, and abnormal vascularity [7]. Cornual pregnancies are diagnosed when a gestational sac is located in the upper lateral uterine horn, surrounded by a thin (<5 mm) myometrial mantle, and separated from the endometrial cavity [6].

This case presented diagnostic challenges due to atypical ultrasound findings. Nevertheless, the clinical team maintained a high index of suspicion and adopted a multidisciplinary approach to manage the case effectively. Histopathological confirmation of placenta increta supported the intraoperative diagnosis [1,2].

## Conclusions

This case represents a rare and life-threatening combination of cornual ectopic pregnancy and placenta increta in a patient without prior cesarean delivery. Early diagnosis, multidisciplinary coordination, and timely surgical intervention were key to achieving a favorable maternal outcome.
